# Survival of Patients Following First Diagnosis of Diabetic Foot Complications: A Nationwide 15-Year Longitudinal Analysis

**DOI:** 10.3389/fendo.2021.801324

**Published:** 2021-12-13

**Authors:** Chia-Hung Lin, David G. Armstrong, Pi-Hua Liu, Cheng-Wei Lin, Chung-Huei Huang, Yu-Yao Huang

**Affiliations:** ^1^ Division of Endocrinology and Metabolism, Department of Internal Medicine, Chang Gung Memorial Hospital, Linkou, Taiwan; ^2^ Graduate Institute of Clinical Medical Sciences, Chang Gung University, Taoyuan, Taiwan; ^3^ College of Medicine, Chang Gung University, Taoyuan, Taiwan; ^4^ Southwestern Academic Limb Salvage Alliance (SALSA), Department of Surgery, Keck School of Medicine of University of Southern California (USC), Los Angeles, United States; ^5^ Clinical Informatics and Medical Statistics Research Center, Chang Gung University, Taoyuan, Taiwan; ^6^ Department of Medical Nutrition Therapy, Chang Gung Memorial Hospital, Linkou, Taiwan

**Keywords:** diabetic foot, peripheral artery disease, cardiovascular diseases, survival, type 2 diabetes, heart failure, amputation

## Abstract

**Background and Aims:**

The long-term survival in people with type 2 diabetes following first diagnosis of diabetic foot complications (FDDFC) is unclear. The object is to evaluate the mortality rate in subjects with type 2 diabetes following FDDFC and the impacts of the major cardiovascular comorbidities.

**Methods:**

Nationwide data were analyzed for subjects with T2D and DFC between 2003 and 2017 according to ICD-9 coding. DFC was defined with the codes of ulcers, infections, or severe peripheral artery disease that required intervention (PAD) to mimic the real world diagnosis. Criteria of FDDFC were preceded by a period without any DFC for at least 5 years. Major cardiovascular comorbidities: established PAD and cardiovascular diseases (CVD: including coronary heart disease (CHD), stroke, or heart failure) before the index date as well as lower-extremity amputations (LEA) at the index episode were analyzed.

**Results:**

Among 300,115 subjects with DFC, a total of 103,396 patients had FDDFC. The mean 5-year survival rate of these subjects was 81.05%. Using subjects without associated major cardiovascular comorbidity as baseline, the adjusted hazard ratios (aHR) were1.43 (95% confidence interval 1.38–1.49) in group PAD-/CVD+, followed by 1.70 (1.59–1.80) in PAD+/CVD- and 1.98 (1.89–2.08) in PAD+/CVD+. The aHR was further increased in patients with PAD who additionally had heart failure (3.77, 3.50–4.05), stroke (2.06, 1.95–2.18), or CHD (1.89, 1.79–2.00). Subjects with PAD rather than other CVD were associated with LEA at FDDFC. Patients with major LEA (above the ankle) at FDDFC episode had lower 5-year survival rate (65.01%) followed by those with minor LEA (72.24%) and without LEA (81.61%).

**Conclusions:**

Cardiovascular comorbidity as well as LEA status at the event of FDDFCs were both associated with patient survival outcomes. Earlier identification of this large population could lead to higher survival rates.

## Introduction

Globally, an estimated 19–34% of people with diabetes are expected to develop foot ulcers in their lifetime (1, 2). Diabetic foot complications (DFC), the leading causes of infection, hospitalization, and amputation outcomes, are readily preventable with early team care (3, 4). However, subjects with DFC are not only vulnerable to lower- extremity amputation (LEA) but are also affected in terms of long-term life expectancy (5–9), with reduction of life expectancy in patients with diabetic foot ulcers being reported in both hospital (5, 7, 9)- and community (7)-based studies at a 5-year survival rate of approximately 55% (8–10). The survival rate in patients with DFC receiving LEA was even worse (10) and poorer than that of patients with common cancers (11). Nevertheless, due to the nature of frequent recurrence in patients with foot ulcers, life expectancy following the first diagnosis of DFC (FDDFC) is difficult to approach in real-world clinical settings.

Cardiovascular disease is the leading cause of death in patients with diabetes (12, 13). Peripheral artery disease (PAD), coronary heart disease (CHD), stroke or heart failure are commonly used as major cardiovascular events in cardiovascular trials in diabetes (14, 15) and are frequently associated as comorbidities in patients with DFC (16, 17). To the best of our knowledge, studies regarding impacts of these major cardiovascular comorbidities on life expectancy in patients with DFC are still lacking. Recently we have reported nationwide trends of DFC in terms of patient characteristics, comorbidities, treatments, and associated LEAs by using the Taiwanese National Health Insurance (NHI) system (18). The Taiwan NHI provides coverage for over 99.6% of the 23 million people in Taiwan (19, 20). Using this NHI database, this study was conducted to evaluate the long-term survival and its associated factors in patients with FDDFC.

## Methods

### Data Source

This retrospective cohort study was conducted using longitudinal claims data from Taiwan’s National Health Insurance Research Database (NHIRD) from 2003 to 2017. The Institutional Review Board of Chang Gung Memorial Hospital, Taiwan (No. 107-2041C1) approved this study. The Health and Welfare Data Science Center was used for accessing diabetes mellitus foot data.

### Study Population

**Figure 1:**
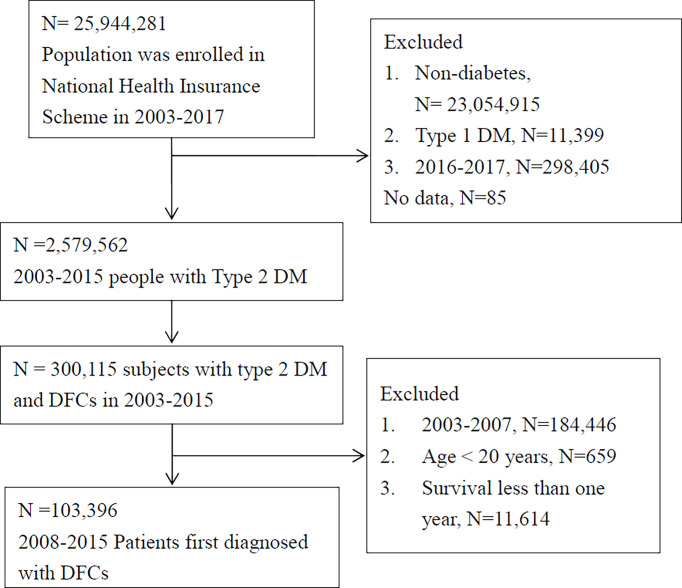
Flowchart of inclusion. Individuals with type 2 DM and first diagnosis of diabetic foot complications (DFC) were included in our analysis after relevant exclusions (DM, diabetes mellitus; PAD, peripheral artery disease; CVD, cardiovascular disease, including non-fetal myocardial infarction, stroke, or heart failure).

The present study employed data collected from 2003 to 2017 from the NHIRD. The Taiwanese government implemented its NHI system in 1995, providing coverage for 95% of the population by 2000, subsequently increasing this to 98% in 2005 and 99.6% of the 23 million people in Taiwan by 2009. Large computerized administrative and claims data sets derived from this program have provided diagnoses, procedures, and prescriptions of inpatient and outpatient records. For this study, patients with type 2 diabetes were identified as those receiving the ICD-9 diagnostic code of 250 (except for 250.01 and 250.03 for type 1 diabetes) at least once during hospital admission or three or more times at an ambulatory clinic in a given calendar year (**Figure 1**). Type 1 diabetic cases were excluded because of the lower number of cases and younger age (20, 21).

### Definition of Diabetic Foot Complications

DFCs include diabetic foot ulcers, foot infections, or severe PAD required for intervention. All diagnoses were based on ICD-9 diagnostic codes or NHIRD procedure codes. Foot ulcers included diagnostic codes 707.06, 707.07, 707.1, 892, and 893. Diabetic foot infections were identified by foot cellulitis and abscess (680.6, 680.7, 681.10, 682.6, and 682.7); osteomyelitis (730.06, 730.07, 730.16, 730.17, 730.26, 730.27, 730.96, and 730.97); and necrotizing fasciitis (728.86, 040.0). Severe PADs were identified by the presentation of gangrene (785.4 and 440.24) or hospitalization for PAD (440.2, 440.3, 440.4, 443.81, 443.89, 443.9, 444.22, 445.02, and 785.4).

### Definition of First Diagnosed DFC

The FDDFC was defined as a DFC diagnosis with no other DFC in the preceding 5 years (mean 8.9 ± 2.3 years in this study). Only the first event of any aforementioned DFC was set as the index date to prevent the overestimation of DFC rates due to the inclusion of patients with multiple foot complications.

### Coding for Comorbidities and Major-CVDs

All the comorbidities were confirmed before or at the index date of FDDFC. The comorbidities including the codes for hypertension (401, 402, 403, 404, and 405), dyslipidemia (272.0, 272.1, 272.2, 272.3, and 272.4), diabetic retinopathy (362 and 250.5), diabetic neuropathy (250.6), diabetic nephropathy (250.4, 585, 586, V42.0, V45.1, V56, 403, and 404), and end-stage renal disease (585.5, 585.6, 586, V45.11, V45.1, V56.X, 403.01, 403.11, 403.91, 404.02, 404.03, 404.12, 404.13, 404.92, and 404.93).

In addition to patients with severe PAD, those who had underlying MI, stroke, or heart failure were included in analyses as a major-CVD comorbidity. The codes included those for nonfatal MI (410 with procedural codes of percutaneous coronary intervention, coronary artery bypass surgery, or thrombolysis therapy), nonfatal stroke (430, 431, 432, 433, 434, 435, 436, and 437.1) and admission for heart failure (428).

### Coding for LEAs

The diagnostic codes V49.70, V49.71, V49.72, V49.73, and V49.74 and procedure codes 84.11 and 84.12 were used for minor LEA (below the ankle), while diagnostic codes V49.75, V49.76, and V49.77 and procedure codes 84.14, 84.15, and 84.17 were used for major LEA (above the ankle). The specific traumatic amputation codes such as 8950, 8962, 8971, 8974, 8977 were excluded from the current study.

### Survival Estimates

Survival time was calculated from the index date of FDDFC to December 31, 2017 or death. The date of death was obtained from the National Death Registry of the Taiwan NHI database. Information of individuals who were alive at the end of the study was collected as right-censored data, and the Kaplan–Meier method was therefore used to calculate the survival time.

### Statistical Analysis

Chi-square test and ANOVA were used to compare the groups of major-CVD before FDDFC. Patients receiving LEA at the FDDFC event were grouped into major, minor and no LEA for comparison. A p-value < 0.05 was considered statistically significant with multivariate Cox proportional hazards regression models used to identify the predictors of survival endpoints, while results were expressed as adjusted hazard ratios (aHR), 95% CIs and forest plots. Cumulative survival curves were plotted using the Kaplan–Meier method and compared using a log-rank test. All calculations were performed with SAS software, version 9.4.

## Results

### Allocation of the Study Population

From year 2003 to 2017, a total of 25,944,281 individuals were included in the nationwide claims data. Subjects without diabetes (n=23,054,915) and patients with type 1 diabetes (n=11,399) were excluded. To ensure the follow-up period for at least two years in this study, subjects with T2D found in the last two years of this study (i.e. years 2016-2017; n=298,405) were further excluded (**Figure 1**). Finally, a total of 2,579,562 subjects with type 2 diabetes and 300,115 claims of patients identified to have DFC were used for analysis. To ensure the “first diagnosis” of DFC, subjects with DFC diagnosis in the first five years of this study (i.e. between 2003 and 2007; n=184,446) were excluded, while an additional 659 patients with age less than 20 years and a further 11,614 patients with survival less than one year were also excluded from the analysis. Eventually, a total of 103,396 subjects with type 2 diabetes were confirmed as FDDFC and ensured to have at least two years of follow-up after FDDFC. For these patients, the mean DFC-free period before the index date was 8.9 ± 2.3 years.

### Patient Characteristics According to Their Associated Major-CVDs

We analyzed the demographics between patients with FDDFC having comorbidity of various components of major cardiovascular diseases (**Table 1**). The 48,166 subjects (46.6%) had neither coding of severe PAD nor other major-CVD (PAD-/CVD-) at the index date. On the other hand, 18,035 (17.44%) had associated PAD and 48,975 (47.36%) had a history of other major-CVD. To analyze the impacts of individual component of CVD, we therefore divided patients into four groups, PAD-/CVD-(N=48,166), PAD-/CVD+ (N=37,165), PAD+/CVD-(N=6,225) and PAD+/CVD+ (N=11,810).

**Table 1:** Demographics of patients at first diagnosis of diabetic foot complications (FDDFC).

	PAD-/CVD-	PAD-/CVD+	PAD+/CVD-	PAD+/CVD+	P-value
	N = 48,166	N = 37,165	N = 6,255	N = 11,810	
Age (mean±SD)	59.91 ± 13.62	69.47 ± 11.9	64.4 ± 13.73	71.66 ± 10.99	<0.001
Age group, n (%)					<0.001
<=50	11922 (24.75)	2405 (6.47)	1001 (16.00)	402 (3.4)	
51-65	19754 (41.01)	11065 (29.77)	2304 (36.83)	3009 (25.48)	
66-80	13133 (27.27)	16513 (44.43)	2123 (33.94)	5656 (47.89)	
>80	3357 (6.97)	7182 (19.32)	827 (13.22)	2743 (23.23)	
Gender, n (%)					<0.001
Male	28557 (59.29)	19870 (53.46)	3700 (59.15)	6493 (54.98)	
Female	19609 (40.71)	17295 (46.54)	2555 (40.85)	5317 (45.02)	
Comorbidities, n (%)					
Hypertension	25131 (52.18)	28728 (77.3)	3548 (56.72)	9229 (78.15)	<0.001
Dyslipidemia	20510 (42.58)	18592 (50.03)	2810 (44.92)	6660 (56.39)	<0.001
Diabetic nephropathy	7287 (15.13)	9790 (26.34)	1401 (22.4)	4548 (38.51)	<0.001
Diabetic retinopathy	5855 (12.16)	6237 (16.78)	1018 (16.27)	2436 (20.63)	<0.001
Diabetic neuropathy	4334 (9.00)	5041 (13.56)	778 (12.44)	1798 (15.22)	<0.001
End-stage renal disease	1369 (2.84)	3261 (8.77)	562 (8.98)	2546 (21.56)	<0.001
LEA, n (%)					
Major LEA	94 (0.2)	83 (0.22)	354 (5.66)	584 (4.94)	<0.001
Minor LEA	403 (0.84)	231 (0.62)	912 (14.58)	763 (6.46)	
No LEA	47669 (98.97)	36851 (99.16)	4989 (79.76)	10463 (88.59)	

PAD, Severe PAD been identified by the presentation of gangrene or hospitalization for intervention.

CVD, Underlying coronary heart diseases, stroke, or heart failure.

LEA, Lower-extremity amputation.

SD, Standard deviation.

Patients without any major-CVD components (PAD-/CVD-) had lowest mean age at 59.91 ± 13.62 years, while patients with both PAD and CVD (PAD+/CVD+) had the highest mean age at 71.66 ± 10.99 years (p < 0.001) (**Table 1**). In addition, patients in the PAD-/CVD+ group were older than those in the PAD+/CVD- group (69.47 ± 11.9 vs. 64.4 ± 13.73 years, p < 0.001). In spite of the presence of PAD, the predominant age group of patients without CVD was younger that those with CVD (51–65vs. 66–80 years, p < 0.001).

The rates of comorbidities with hypertension, dyslipidemia, diabetic nephropathy, retinopathy, neuropathy, and end-stage renal disease (ESRD) increased along with the presence of CVD (p < 0.001). The rate of LEA was mainly associated with the presence of PAD but not for the other CVD (p < 0.001).

### Impacts of Associated Major-CVD on Survival

**Figure 2:**
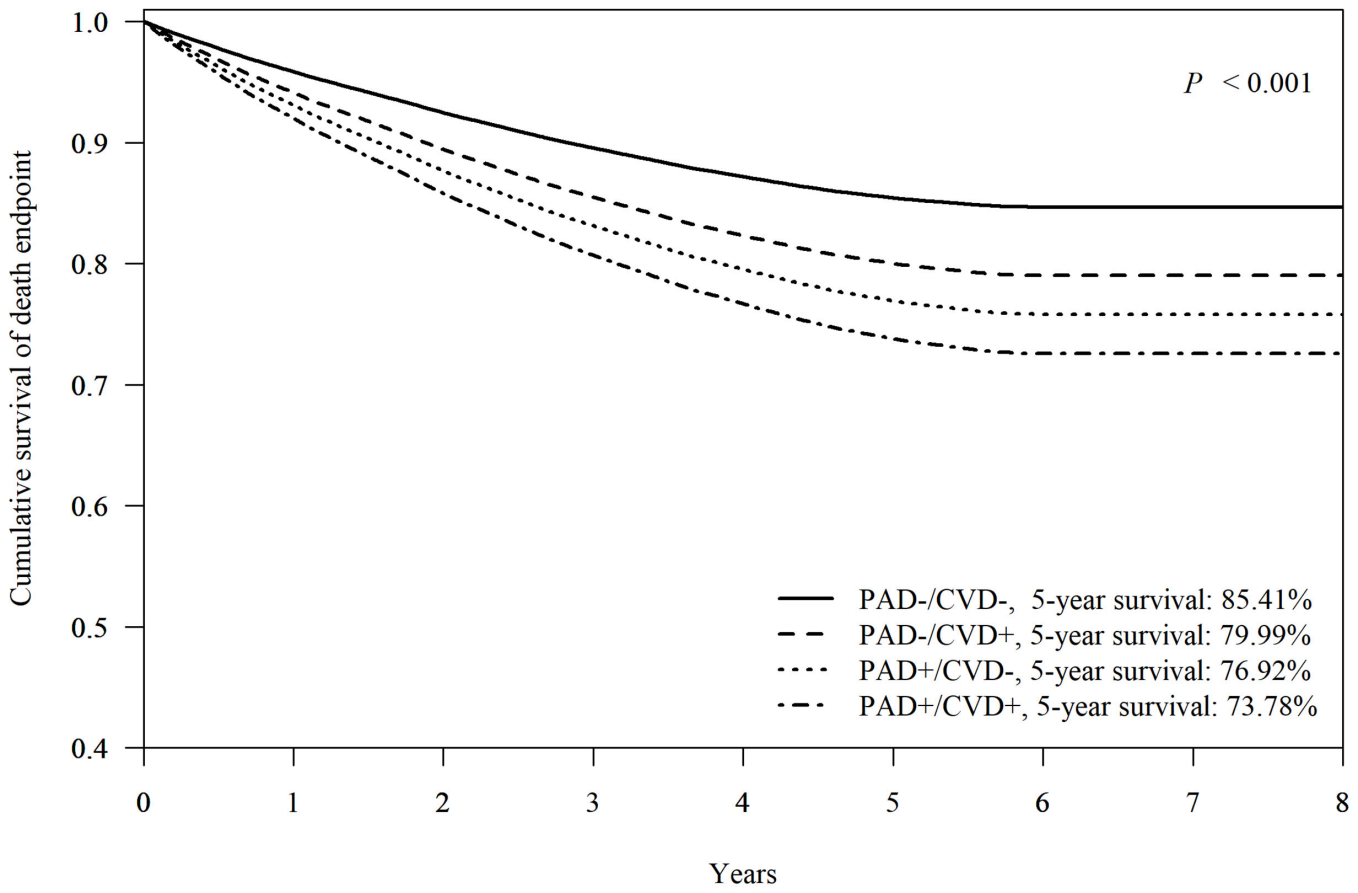
Cox-adjusted survival analysis of patients with or without peripheral artery disease (PAD) or other major-cardiovascular disease (CVD, including non-fetal myocardial infarction, stroke, or heart failure). Adjusted factors include age, sex, hypertension, dyslipidemia, diabetic nephropathy, diabetic retinopathy, diabetic neuropathy, and end-stage renal disease.

Among 103,396 patients with FDDFC, the mean 5-year survival rate was 81.05%.When neither PAD nor CVD was present, the 5-year survival rate for the patients with at first diagnosis of DFCs was 85.41% (**Figure 2**). The presence of either CVD or PAD reduced the 5-year survival rate to 79.99% and 76.92% respectively. The 5-year survival rate was only 73.78% for patients with both PAD and CVD (p < 0.001). The difference in comorbidity presentation led to different mortality outcomes in patients with diabetic foot.

### Impacts of Individual Component of CVD on Survival

**Figure 3:**
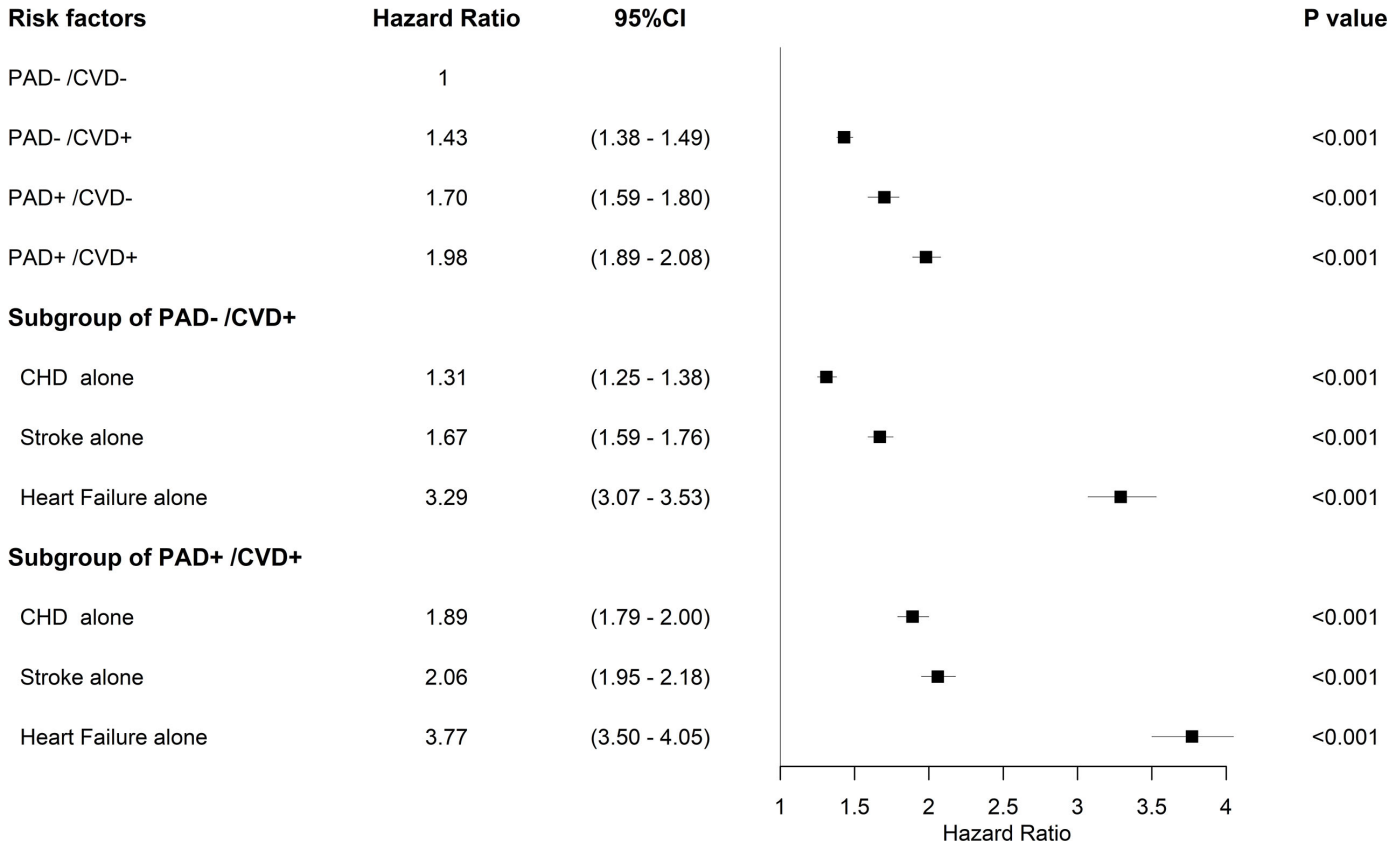
Forest plots for mortality and risk factors adjusted for age group, sex, hypertension, dyslipidemia, diabetic nephropathy, diabetic retinopathy, diabetic neuropathy, and end-stage renal disease in the subgroups.

When using the mortality rate of group PAD-/CVD-as a reference, the aHR (95% CI) for PAD-/CVD+, PAD+/CVD-, and PAD+/CVD+ was 1.43 (95% CI 1.38–1.49), 1.70 (95% CI 1.59–1.80), and 1.98 (95% CI 1.89–2.08) respectively, indicating the impacts of association with PAD or other CVD at FDDFC on survival. The combination of PAD and CVD however, showed additional impacts on survival. Analysis of the individual components of CVD revealed that heart failure had the highest aHR (3.29, 95% CI 3.07-3.53), followed by stroke (1.67, 95% CI 1.59-1.76) and coronary heart disease (CHD) (1.31, 95% CI 1.25-1.38) in subjects without PAD (**Figure 3**). Moreover, in patients with PAD at FDDFC, the risk was further increased with a history of heart failure (3.77, 95% CI 3.50–4.05), stroke (2.06, 95% CI 1.95–2.18), or CHD (1.89, 95% CI 1.79–2.00) (p < 0.001), as indicated in **Figure 3**.

### Impacts of Major-CVD on LEAs

**Table 1** also demonstrates the limb status of FDDFC. Subjects had 98.97% and 99.16% chance of limb preservation if not having major-CVD or having CVD other than PAD respectively. On the contrary, subjects with PAD+/CVD- had 5.66% major LEA and 14.58% minor LEA. For subjects with PAD+/CVD+, the major- and minor- LEA were found to be 4.96 and 6.46% respectively.

### Impacts of LEAs at FDDFC on Survival

**Figure 4:**
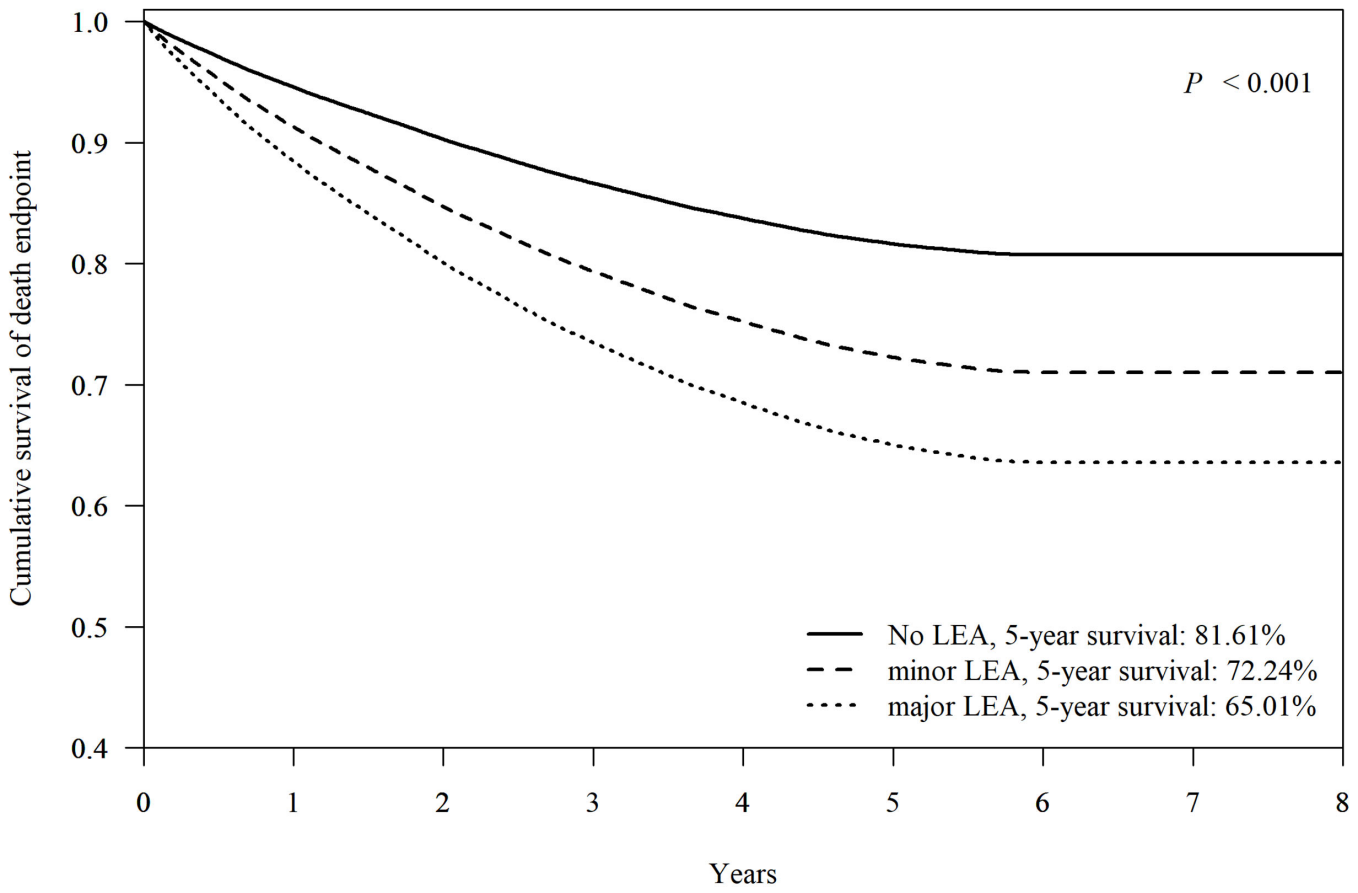
Cox-adjusted survival analysis of patients without lower-extremity amputation (no LEA), minor- (below the ankle) or major- (above the ankle) LEA. Adjusted factors include age, sex, hypertension, dyslipidemia, diabetic nephropathy, diabetic retinopathy, diabetic neuropathy, and end-stage renal disease.

**Figure 4** demonstrates Cox-adjusted survival analysis of patients with or without LEA at FDDFC. The patients with any LEA at the index episode had the lower 5-year survival rate than those without LEA (69.63% vs. 81.61% respectively, p < 0.001). The individual 5-year survival rates of patients with major LEA, and minor LEA were 65.01% and 72.24% (p < 0.001).

The mean age of subjects having major LEA was older than those having minor or no LEA (68.95 ± 11.93 vs. 63.75 ± 12.73 and 64.94 ± 13.7 years respectively, p < 0.001) (**Table 2**). It was noted that for those patients having major LEA, the highest incidence was found in the 66- to 80-year-old age group while for those having minor LEA, the highest age group was for 51-65-year-olds. The male gender was more predominant in patients with FDDFC, which was more prominent than in those having major LEA. The comorbidities of hypertension, nephropathy and ESRD were significantly increased in the subjects having major LEA.

**Table 2:** Demographics of patients with or without LEA.

	Major LEA	Minor LEA	No LEA	P-value
	N = 1,115	N = 2,309	N = 99,972	
Age (mean±SD)	68.95 ± 11.93	63.75 ± 12.73	64.94 ± 13.7	<0.001
Age group				<0.001
<=50	63 (5.65)	354 (15.33)	15313 (15.32)	
51-65	375 (33.63)	935 (40.49)	34822 (34.83)	
66-80	465 (41.7)	788 (34.13)	36172 (36.18)	
>80	212 (19.01)	232 (10.05)	13665 (13.67)	
Gender				<0.001
Male	676 (60.63)	1477 (63.97)	56467 (56.48)	
Female	439 (39.37)	832 (36.03)	43505 (43.52)	
Comorbidities, n (%)				
Hypertension	714 (64.04)	1326 (57.43)	64596 (64.61)	<0.001
Dyslipidemia	441 (39.55)	961 (41.62)	47170 (47.18)	<0.001
Diabetic nephropathy	405 (36.32)	712 (30.84)	21909 (21.92)	<0.001
Diabetic retinopathy	180 (16.14)	503 (21.78)	14863 (14.87)	<0.001
Diabetic neuropathy	119 (10.67)	304 (13.17)	11528 (11.53)	0.034
End-stage renal disease	235 (21.08)	367 (15.89)	7136 (7.14)	<0.001

## Discussion

To the best of our knowledge, this is the first study to explore the natural survival of patients from first diagnosis of DFC in a large population, reporting the predictive value of major cardiovascular comorbidity as well as limb status with long-term survival in patients with FDDFC.

The 5-year survival in patients with FDDFC of this study was 81.05%, which was much better compared to 40-50% reported for treatment of diabetic foot diseases (22–24). This might be due to the frequently recurring nature of diabetic foot diseases and the current lack of awareness of use of DFC as a high-risk group in general practice. The patients recruited in the previous reports were not limited to the first diagnosed DFC. Otherwise, the patients with foot cellulitis were also included in the current study, which represented the early condition of DFC. Compared to the recurrence ulcers, patients with first diagnosed DFC may present less severe or lower grade of DFC (including ulcers, infection or perfusion). In addition, better chance of wound healing may occur at their first or earlier event because subjects may have better health status as well as associate with less diabetic comorbidities. Because the first diagnosed DFC was the main study objects, we enrolled these patients to make the condition compatible to the real world practice. It also could be the cause of high survival rate in this study. Earlier identification of these patients, as shown in this study, might provide strategies to prolong survival or limb preservation.

The risk of mortality from cardiovascular diseases is four-fold higher among patients with type 2 diabetes than among individuals without diabetes (25). Recent large-scale cardiovascular outcome trials have reported mortality in patients with type 2 diabetes and established cardiovascular comorbidity was up to 8.3%–9.6% within 3–4 years (14, 15). In the current study, we further analyzed patients with FDDFCs and disclosed that the established PAD as well as other major-CVD corresponded to a significant reduction in 5-year survival rate from 85.41% to 73.78%. Patients with DFC share common pathological conditions, including atherosclerosis cardiovascular diseases, which are highly associated with PAD (26). PAD itself is regarded as a manifestation of systemic atherosclerosis rather than only a localized disease and one that, similar to other cardiovascular diseases, results in higher mortality in patients with type 2 diabetes (27). Previous reports have shown the common existence of PAD in patients with diabetes and its independent association with all-cause mortality and cardiac mortality (27, 28).

Compared to other major-CVD, the presence of PAD resulted in a higher risk of LEA during the treatment of FDDFCs. Subsequently, a mortality risk no less than that of established other CVD in long-term survival been observed, with a combination of PAD and additional CVD leading to higher morbidity and mortality.

In this study, patients with FDDFCs and heart failure had a three-fold higher risk of mortality than those without association with heart failure. Patients with diabetes and heart failure were reported to have a higher cumulative rate of mortality at 9.4% (29). A study conducted in Italy reported that the mortality rate was increased by 30.8% among patients with diabetes who had heart failure and foot ulcers (30). As a result, the presentation of heart failure requires clinical awareness.

A higher prevalence of ischemic stroke has been reported in patients with DFCs (31). Nevertheless, the impact of ischemic stroke on survival in patients with DFCs is still unclear. In the current study, it was found that the adjusted HR for mortality was increased in the presence of stroke, and further increase in mortality risk has been observed when concomitant with a PAD. The interaction of PAD and ischemic stroke has been little mentioned in previous outcome analyses (32).

The prevalence and overall risk of mortality of patients with DFCs were significantly higher among male patients in a previous report (33). In the current study, the trend of male predominance in DFD was consistent from the group with the lowest risk that had both negative PAD and CVD presentation to the group with highest risk having both positive PAD and CVD presentation; however, the percentage of the female gender also increased parallel to the presentation of PAD or CVD in our study. Accordingly, the DFCs should be aggressively treated irrespective of gender.

Patients with DFCs have a variety of clinical presentations and morbidity as well as mortality outcomes. A higher mortality rate has been reported in patients with diabetes and major LEA than those with minor LEA (10). In our previous report, limb status at treatment and BMI were independent factors of survival in patients with DFCs (8). For clinical application, outcome prediction of patients with first diagnosis of DFCs is worth further investigation. In the current study, patients with FDDFCs were further analyzed from the NHIRD and it was determined that major LEA corresponded to a drastic reduction in 5-year survival rate from 81.61% to 65.01%. The risk of death has been known to increase after LEA in patients with diabetes (34). Our current study has further shown that the mortality rate was higher in major than minor LEA of patients with FDDFC. Older age, higher percentages of hypertension, nephropathy and ESRD were the characteristics among these patients. This study is the first large-scale survey for proof of the crucial role limb preservation plays for such patients with first diagnosis of DFCs.

### Limitations

This was a nonrandomized, retrospective, observational study, and thus selection bias was possible despite comprehensive propensity score-matching. This study used Taiwan NHIRD to exemplify a population-level data source for generating real-world evidence. Nevertheless, like with all claims databases, there have been some validity concerns of the accuracy of diagnosis codes and issues around unmeasured confounders. Patients with diabetic foot belong to a high-risk group for cardiovascular disease however, and these confounding factors might have been minimized by our large cohort size and adequate data clearance of five years as well as the follow-up period of at least one year. Medication was not considered because it would have further complicated the already complex model and led to a low matching rate between groups. Though some new anti-diabetic drugs e.g. sodium-glucose cotransporter 2 inhibitors (SGLT2i) may show interests for CV and amputation outcomes, the analysis was not performed due to this class of drugs launched in Taiwan after 2017. Data of glycemic control were lacking in this database, although it is a strong confounding factor in mortality. The diagnostic codes cannot totally represent the disease condition, especially in detailed information, like the wound size, peripheral perfusion or the successful rate of vascular interventions. Since the limitation in the classification of DFC, we did not do the subgroup analysis. Some details of diabetic foot, including PEDIS classification for the wound, could not be distinguished by ICD-9 codes, so diabetic foot status might have affected patient outcomes. But for the majority of patients with diabetic foot, we could still assess the effects of comorbidities on mortality.

## Conclusion

The earlier identification and therefore diagnosis of this large population might explain higher survival before compared to previous studies. The comorbidities of major CVD especially heart failure deeply affected long-term survival besides LEA. Earlier identification of diabetic foot complications with strategies provided might have a positive impact on 5-year mortality.

## Data Availability Statement

The original contributions presented in the study are included in the article/supplementary material. Further inquiries can be directed to the corresponding author.

## Ethics Statement

The studies involving human participants were reviewed and approved by The Institutional Review Board of Chang Gung Memorial Hospital, Taiwan (No. 107-2041C1). Written informed consent for participation was not required for this study in accordance with the national legislation and the institutional requirements.

## Author Contributions

C-HL wrote the manuscript and researched data. Y-YH researched data and contributed to discussion. DA contributed to discussion and reviewed/edited the manuscript. C-WL and C-HH researched data. P-HL and C-HL reviewed/edited the manuscript. Y-YH is the guarantor of this work, has full access to all the data in the study, and takes responsibility for the integrity of the data and the accuracy of the data analysis. All authors contributed to the article and approved the submitted version.

## Funding

This research was supported by the Chang Gung Medical Research Program grant (Grant Number: CIRPD1D0032, CMRPD1H0531, CORPG5F0011, CFRPG3K0041, CMRPG3H0941, CMRPG3H0942, CMRPG3H0943). This study was partially supported by National Institutes of Health, National Institute of Diabetes and Digestive and Kidney Diseases Award Number 1R01124789-01A1. The funders had no input into any aspect of the design and management of this study.

## Conflict of Interest

The authors declare that the research was conducted in the absence of any commercial or financial relationships that could be construed as a potential conflict of interest.

## Publisher’s Note

All claims expressed in this article are solely those of the authors and do not necessarily represent those of their affiliated organizations, or those of the publisher, the editors and the reviewers. Any product that may be evaluated in this article, or claim that may be made by its manufacturer, is not guaranteed or endorsed by the publisher.
